# The Genomic Variation in the Aosta Cattle Breeds Raised in an Extensive Alpine Farming System

**DOI:** 10.3390/ani10122385

**Published:** 2020-12-12

**Authors:** Maria Giuseppina Strillacci, Mario Vevey, Veruska Blanchet, Roberto Mantovani, Cristina Sartori, Alessandro Bagnato

**Affiliations:** 1 Department of Veterinary Medicine, Università degli Studi di Milano, Via dell’Università 6, 20133 Milano, Italy; maria.strillacci@unimi.it; 2 Associazione Nazionale Bovini di Razza Valdostana, Fraz. Favret, 5, 11020 Gressan, Italy; direttore@anaborava.it (M.V.); veruska.blanchet@gmail.com (V.B.); 3 Department of Agronomy, Food, Natural Resources, Animals and Environment (DAFNAE), Università degli Studi di Padova, Viale dell’Università 16, 35020 Legnaro, Italy; roberto.mantovani@unipd.it (R.M.); cristina.sartori@unipd.it (C.S.)

**Keywords:** Aosta cattle breeds, Runs of Homozygosity, inbreeding, ROH, autochthonous breeds

## Abstract

**Simple Summary:**

Genetic variability among native cattle breeds can disclose the important features that make a population adapted to harsh environments. The Aosta cattle breeds have been raised and selected for centuries to be farmed in a mountain environment, characterized by a semi-intensive system, i.e., summer pasture with winter recovery on the farms. To disclose the genomic variation and its association with known genes, it is important to genetically characterize these breeds.

**Abstract:**

The Aosta Red Pied (Valdostana Pezzata Rossa (VRP)), the Aosta Black Pied (Valdostana Pezzata Nera (VBP)) and the Aosta Chestnut (Valdostana Castana (CAS)) are dual-purpose cattle breeds (meat and milk), very well adapted to the harsh environmental conditions of alpine territories: their farming is in fact characterized by summer pasture at very high altitude. A total of 728 individuals were genotyped with the GeenSeek Genomic Profiler® (GGP) Bovine 150K Illumina SNP chip as a part of the DUALBREEDING-PSRN Italian-funded research project. The genetic diversity among populations showed that the three breeds are distinct populations based on the F_ST_ values, ADMIXTURE and Principal Component Analysis (PCA) results. Runs of Homozygosity (ROH) were obtained for the three populations to disclose recent autozygosity. The genomic inbreeding based on the ROH was calculated and coupled with information derived from the *F* (inbreeding coefficient) and F_ST_ parameters. The mean F_ROH_ values were low: CAS = 0.06, VBP = 0.05 and VRP = 0.07, while the average *F* values were −0.003, −0.01 and −0.003, respectively. The annotation and enrichment analysis, performed in the identified most frequent ROH (TOP_ROH), showed genes that can be linked to the resilience capacity of these populations to harsh environmental farming conditions, and to the peculiar characteristics searched for by farmers in each breed.

## 1. Introduction

Animal genetic resources play an important role in local economies and in maintenance of territories and landscapes [1]. Among the large number of autochthonous cattle populations in Italy, the Aosta breeds play an important role for the Aosta valley, located in the northwest Alpine territories of Italy [2]. In addition to their milk and meat production, their economic value is also related to the farming activity itself, closely linked to the use of local territories: farming activity, in fact, allows to maintain the territory and the environment and to keep alive the strong cultural and societal value that these breeds and their farming represent.

Aosta breeds are the Aosta Red Pied (Valdostana Pezzata Rossa (VRP)), the Aosta Black Pied (Valdostana Pezzata Nera (VBP)) and the Aosta Chestnut (Valdostana Castana (CAS)). All three are dual-purpose cattle breeds and possess a considerable milk production (2019 average production per lactation: 3000 kg of milk for CAS and VBP and 4000 for VRP) in proportion to their body size (average adult live weight of 550 kg for males and 400 kg for females). Even if their production selection goal includes milk and meat, their capacity to adapt to the harsh alpine environment, i.e., their functionality, has been strongly pursued by farmers for decades [3]. Additionally, the active mating scheme in these populations, done for decades, was implemented in order to avoid loss of genetic variability while applying some directional selection for milk and meat [4]. The summer pastures occurring at high altitudes, up to 2500 m above sea level, challenges the cow’s functionality and capability to cope with severe environmental conditions. These include feeding with fresh grass, ability to walk and climb steep territories and resisting harsh climates. The alpine summer pasture is of central economic value for the Aosta Valley region, as this practice maintain territories and landscapes, making them usable for tourism and, as such, closely linked to the local economy. CAS and VBP are appreciated by farmers, also for their particular vitality and impetuosity, which it takes the form of a dominant behavior within the herd. For decades in the Aosta valley, annual tournaments have been organized among the cows of the CAS and VBP cattle from different herds (Batailles des Reines) and weekly disputed from March to October [5]. The innate instinct for the territoriality and hierarchical dominance of the cows translates first into a non-ferocious ritual of combat, where dominance of a cow over the other is expressed in knock-out battles, leading then to the awarding of the title of Queen of the Valley in a final contest held in Aosta at the “Arena Croix Noir”. Farmers give particular care to select Aosta Chestnut cattle for their temperament [6], as the cultural value of the “*Battailes des Reines*” has a central role for farmers and the Aosta valley’s cultural value and sociality [1]. Nowadays, CAS and VBP can be considered as three purpose breeds because the fighting ability is a selection criteria included in the IRCMC (Indice Resa Casearia, Muscolosità, Combattività—Cheese yield, Muscularity and Combativeness Index), the official selection index of the two breeds [7].

The availability of wide genome SNP genotypes on a large scale in cattle made it possible to obtain a detailed picture of the breeds’ genetic diversity across the genome, to investigate genomic variation among populations and to relate their genomic structure to the occurring selection that is undergoing in cattle populations. Runs of Homozygosity (ROH) are long tracts of homozygous DNA that were firstly identified in human populations by [8]. McQuillan et al. [9] used ROH to investigate genomic variation among European populations and used ROH to propose a new genomic inbreeding coefficient. They compared different coefficients of inbreeding, finding the largest correlation between genomic inbreeding (F_ROH_) and the inbreeding coefficient calculated by genealogical information. Several authors used ROH to explore genomic variation in cattle [10,11,12,13]. ROH can be used to detect recently occurring autozygosity, generated when related individuals are mated and which discloses the genomic regions under selection, including putative candidate genes. Curik et al. [14] discussed the use of ROH and F_ROH_ to identify hotspot regions (i.e., islands), where the frequency of ROH is high, and coldspot regions, where no ROH is found (i.e., deserts).

The nationally funded project DUALBREEDING (PSRN National Program of Rural Development 2017–2020 by Italian Ministry of Agriculture) had among its objectives the disclosure of genomic variation across Aosta breeds and its relationship with occurring selection. Among the activities of DUALBREEDING, all males and a part of the female population have been analyzed with SNP chips at a 150K density providing as such a solid reference genotype database.

The aim of this study was to map ROH in the three Aosta breeds using the 150K SNP chip genotypes available from DUALBREEDING. A further objective was to calculate the F_ROH_ based on the ROH and compare it with information derived from *F* (the inbreeding coefficient). Additionally, we aimed to investigate the genetic variability across breeds using Wright’s F statistics, a Principal Component Analysis and ADMIXTURE analysis. Finally, we aimed to annotate the genes mapped in the ROH in order to disclose the common and proprietary regions under selection in the three Aosta breeds.

## 2. Materials and Methods

### 2.1. Ethics Statement

This study did not require approval from the Animal Care and Use Committee.

### 2.2. Sampling and Genotyping

The Associazione Nazionale Allevatori Bovini di Razza Valdostana (A.N.A.Bo.Ra.Va.) provided genotypes of 728 individuals—male and females (CAS, 297; VBP, 153; and VRP, 278)—obtained with the GGP Bovine 150K Illumina SNP chip. The sampling of the individuals was homogeneous across breeds regarding their population structure and reflects their actual population size. The genotyping is part of the national project DUALBREEDING, a pluriannual effort funded by the EU EAFRD and by the Ministry of Agriculture of Italy aimed at maintaining the biodiversity in cattle populations. The SNP genotypes were mapped on the ARS-UCD1.2 bovine reference genome. Out of the genotypes available on the SNP chip, those with a sample and marker call rate ≤0.90, without chromosomal position and on non-autosomal chromosomes, were deleted, leaving a total of 128,180 SNP markers for subsequent analyses.

### 2.3. Aosta Breeds Diversity Performed by PCA, F_ST_ and ADMIXTURE

Genetic diversity within and among breeds was determined through a Principal Component Analysis (PCA) and estimating the pairwise Fixation Index through the pipelines implemented in Golden Helix (SVS) 8.8.4 software (Golden Helix Inc., Bozeman, MT, USA). The Fixation Index—equivalent to Wright’s F-statistic F_ST_—is a measure of the total genetic variance that can be explained by the population structure; in other words, F_ST_ estimates the genetic divergence among subpopulations. The F_ST_ was estimated for three pairs of breed combinations (CAS vs. VBP, CAS vs. VRP, and VBP vs. VRP). The expected (He) and observed (Ho) heterozygosity values were also calculated for each breed.

Determination of the most probable number of ancestral populations was obtained with ADMIXTURE v. 1.3.0 software [15]. ADMIXTURE was run from K = 2 to K = 6 and the optimal number of clusters (K-value) was determined as the one having the lowest cross-validation error. The R script suggested by the ADMIXTURE procedure was used to perform a graphical representation of the ADMIXTURE results. PCA, ADMIXTURE and Wright’s F-statistic were performed on the pruned SNP dataset (83,776 markers, resulted by using r^2^  >  0.5 in a 50-SNP sliding windows in SVS software) in order to reduce the impact of the SNP ascertainment bias from linkage between loci.

### 2.4. F_ST_ Analysis at Marker Level

In order to identify the genome-wide patterns under selection, the outlier loci approach, based on the F_ST_ estimated for each SNP (marker-based F_ST_), was also used and obtained with SVS on the same three breed pair combinations. Genomic regions can be considered being under different (positive) selection in a pair comparison if they contained a high proportion of highly differentiated SNPs based on the F_ST_ values (deviation from neutral loci with an F_ST_ threshold of 0.5) [16]. Differentiated SNPs with an F_ST_ > 0.5 were considered to be under different selection.

### 2.5. Runs of Homozygosity Detection

ROH analyses were performed separately for each population, using SVS software. No linkage disequilibrium (LD)-based pruning was performed and, as in [17], the minimum ROH length was set to 1 Mb to avoid the detection of short and common ROH across the genome due to LD. The ROH were defined setting a minimum of 1000 Kb in length and 60 homozygous consecutive SNPs. In addition, no heterozygote SNPs and no missing SNPs were allowed in the ROH, and a maximum gap between the SNPs of 1000 Kb was set in order to assure that the SNP density did not affect the ROH. ROH were also grouped into 5 classes of length in each set of ROH: <2 Mb, 2–4 Mb, 4–8 Mb, 8–16 Mb and >16 Mb. Descriptive statistics of the ROH were calculated across individuals in each Aosta breed. The genomic regions with the highest frequency of ROH (TOP_ROH) were identified by selecting the top 1% SNPs with the largest occurrence (TOP_SNPs).

### 2.6. Gene Functional Analysis

The full gene set (*Bos taurus*: Annotation Release 105) was downloaded from NCBI [18] and genes were catalogued within the TOP_ROH using the intersectBed command of BEDTools [19]. In addition, the position of all TOP_SNPs with respect to the annotated genes in the TOP_ROH was identified. The SNPchiMp online database [20] was used to convert the Illumina SNP name to the SNP rsID, the unique SNPs code recognized by the Ensembl Variant Effect Predictor (VEP) [21], which was employed to annotate all the TOP_SNPs. Only genes with official “gene name ID” and LOC genes associated with a protein-coding gene name (excluding uncharacterized ones) were considered. A gene ontology (GO) functional annotation and KEGG pathway analyses were performed using DAVID Bioinformatics Resources, version 6.8 [22].

In addition, bovine QTL, available from the “AnimalQTLdb” database (Cattle–ARS-UCD1.2 genome assembly), were catalogued into the TOP_ROH by overlapping [23]. The same SNP annotation approach was applied for differentiated SNPs with an F_ST_ > 0.5.

### 2.7. Inbreeding Coefficients

Identity-by-descent (IBD) was obtained in order to assess the quality of the dataset, to identify the potential sample replicates and to estimate pair-wise comparison of relatedness (comprising first-degree relatives). IBD was calculated using SVS software on pruned SNPs.

SVS software also provided the individual’s inbreeding coefficient *F* [24], which was calculated for each breed. The *F* values ranged from −1 to +1, representing an excess of heterozygosity and an excess of homozygosity, respectively, where an *F* value equal to 0 denotes the Hardy–Weinberg equilibrium across all markers. To compare inbreeding across breeds, the average *F* value was calculated for each breed.

In addition, the inbreeding coefficient based on the ROH (F_ROH_) for each sample and for the three breeds was calculated separately, considering the following formula [8]:

F_ROH_ = L_ROH_/L_AUT_, (1)

where L_ROH_ is the total length of all the proper ROH of an individual, and L_AUT_ is the specified length of the autosomal genome covered by SNPs (2,487,082,459 bp).

## 3. Results

### 3.1. Aosta Breeds’ Diversity

**Figure 1:**
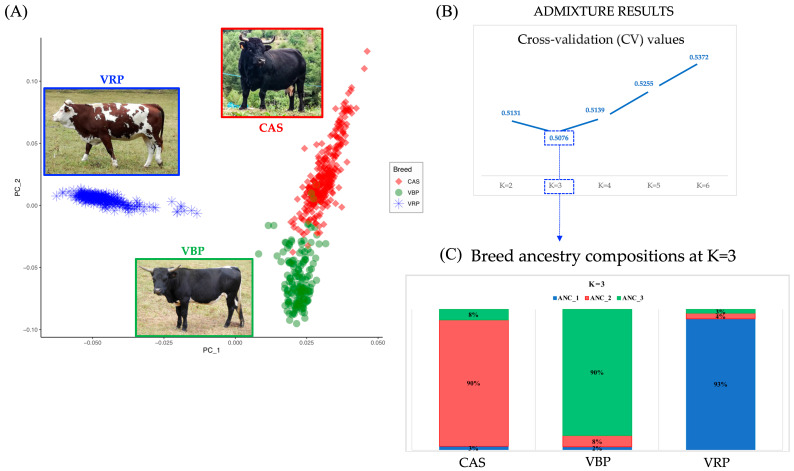
(**A**) PCA on Aosta Red Pied (Valdostana Pezzata Rossa (VRP)—blue stars), the Aosta Black Pied (Valdostana Pezzata Nera (VBP)—green dots) and the Aosta Chestnut (Valdostana Castana (CAS)—red diamonds) samples. Eigenvalues: PC_1 = 16.46; PC_2 = 5.12. (**B**) ADMIXTURE cross-validation error values (CV) plotted at *K* from 2 to 6: lowest CV values were identified at K = 3. (**C**) Proportion of identified ancestral populations (ANC), calculated as the average of the genetic ADMIXTURE score within a population at K = 3. Pictures by M.G. Strillacci.

The effective number of polymorphic SNPs (number of SNPs in which at least one heterozygous individual was identified) represented up the 94% of the total SNPs of all three breeds. The He and Ho values were similarly low among breeds: CAS (0.344 and 0.342), VBP (0.345 and 0.341) and VRP (0.339 and 0.338). The Principal Component Analysis (PCA) result is displayed in Figure 1A. On the first principal component (PC_1) (eigenvalue of 16.46), a clear separation of VRP from CAS and VBP is observable. On the other hand, the PC_2 principal component (eigenvalue of 5.12) allowed to separate the CAS and VBP breeds. Samples belonging to these two breeds appear to be two very close groups, showing partial overlapping.

These results are confirmed by the pairwise breed comparisons using F_ST_, showing that although there is a very low level of genetic differentiation, there is a clear and similar distinction between VRP vs. CAS (F_ST_ = 0.052) and VRP vs. VBP (F_ST_ = 0.050), and a close relationship between CAS vs. VBP (F_ST_ = 0.019). The average lower and upper 95% confidence interval differences from the pairwise breed F_ST_ values were 0.0003 and 0.0008, respectively.

To investigate the ancestry composition of the Aosta breeds, ADMIXTURE analysis was run for values of possible ancestors (K) ranging from 2 to 6. The lowest CV value was obtained at K = 3 (Figure 1B). At this K value, all three breeds appear to be mostly unique populations, represented by an ancestral genetic group in a proportion larger than 90% (Figure 1C). At K = 2, CAS and VBP belonged to a unique ancestral genetic group, while VRP to a different one.

### 3.2. F_ST_ at Marker Level, Gene Annotation and Gene Functional Analyses

The distribution of F_ST_ values, calculated for each marker across all chromosomes for each pair of comparison, is shown in Figure S1. We consider the SNPs’ allelic frequency as differentiated, and then under a possible different positive selection, when the F_ST_ value is >0.5 (above the redline threshold in Figure S1). A total of 53 differentiated SNPs was from the VRP_VBP breeds’ comparison. Forty-one SNPs were mapped in the intergenic regions and 12 in the intronic regions (*CORIN, FIP1L1, LNX1, CLOCK, PIEZO1, CBFA2T3, TMEM104* and *DNAJC12*; Table S1, Sheet 1). If we consider the VRP_CAS breeds’ comparison, 34 SNPs were differentiated markers, of which 29 were annotated in intergenic regions, one in the 3_prime_UTR_variant region of *PDGFRA* gene and four in the intronic positions of the *KIT* (*n* = 1) and *CLOCK* (*n* = 3) genes (Table S1, Sheet 2). No differentiated SNPs were identified for the CAS_VBP breeds’ comparison. There were no significant GO terms and KEGG pathways from the DAVID database for the genes mapped in the differentiated regions.

### 3.3. Runs of Homozygosity Detection

**Figure 2:**
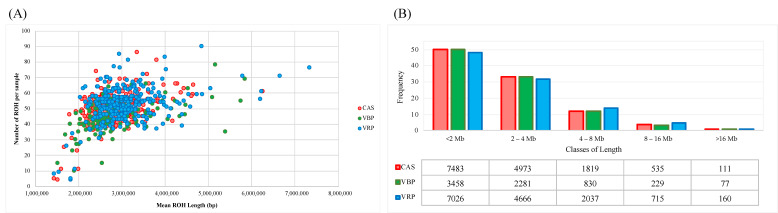
Graphical representation of the Runs of Homozygosity (ROH) statistics: (**A**) relationship between the number and mean total length of the ROH in each sample/breed; (**B**) frequencies and counts of the ROH for each class of length calculated within each breed.

The ROH were identified in all 728 individuals of the three Aosta breeds, for a total of 36,400 homozygous regions. At the individual level, the average numbers of ROH per animal were 50, 45 and 53 for CAS, VBP and VRP, respectively (Table 1), with a total mean ROH length of 2.87, 2.93 and 3.15 Mb, as shown in Figure 2A, which graphically represents the relationship between the ROH counts and the averaged total length of the ROH for each individual.

Differences among animals were also found considering the total length of the genome covered by the ROH (sum of all ROH per animal): 6.10–381.55 Mb for CAS, 19.02–403.27 Mb for VBP and 7.31–557.46 Mb for VRP samples (data not shown).

The ROH were identified for all classes of length and, generally, the lower coverages were in concordance with a low number of regions per samples. Although shorter regions (<2 Mb) are the most frequent classes of length (about 50%; Figure 2B), the proportion of the genome covered by them was relatively small (averaged total length per samples around 1.49 Mb) in all three breeds. Contrariwise, accounting for a small number of ROH per sample, an ROH larger than 16 Mb (CAS and VBP, from 1 to 6; VRP from 1 to 9) covered a wider region of the genome: an ROH >16 Mb was identified in a total of 26%, 30% and 34% of the CAS, VBP and VRP samples, covering up to 5.8%, 6.25%, and 11.3% of their autosome genome.

An ROH was found on all chromosomes and no evident relationship between the chromosomes’ length and mean ROH length was observed: the graphical representation of the ROH frequencies on autosomes together with the mean ROH coverage length calculated for each chromosome is shown in Figure S2. The mean length values of the ROH mapped on autosomes in CAS were more uniform with respect to the ones calculated for VBP and VRP.

**Figure 3:**
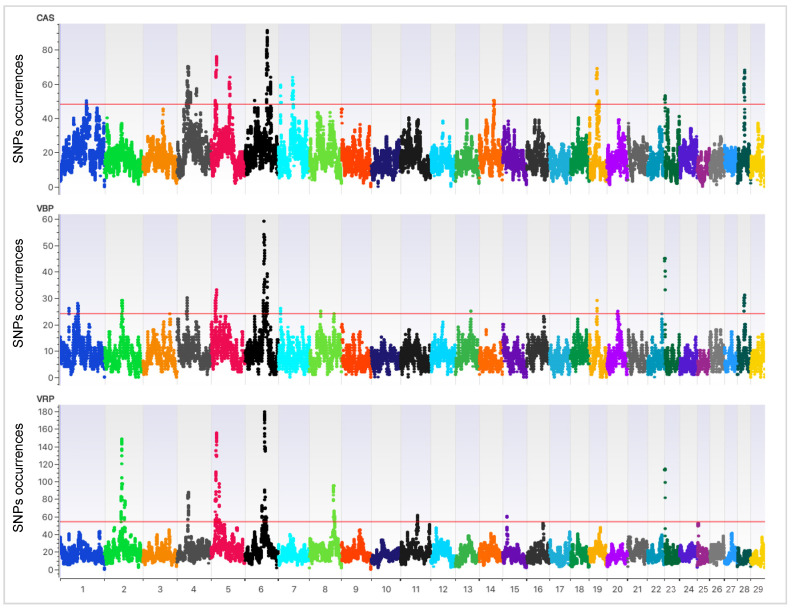
SNP occurrences for CAS, VBP, and VRP. The TOP_ROH were defined by the top 1% SNPs (TOP_SNPs) located above the threshold redlines set at the SNP occurrence values of 48, 24 and 54 for CAS, VBP and VRP, respectively.

In order to explore the effect of selection on the Aosta breeds’ genome, the TOP_ROH regions were considered. The TOP_ROH regions are those located above the redline threshold, as shown in Figure 3 for each breed. The SNP occurrences defining the thresholds were 48, 24 and 54 for CAS, VBP and VRP, respectively. As shown in Figure 3, the genomic distribution of TOP_ROH is clearly non-uniform across autosomes for all three cattle breeds.

TOP_ROH and the annotated genes are reported in Table 2, Table 3 and Table 4 for CAS, VBP and VRP, respectively.

A total of 18 TOP_ ROH on 9 chromosomes (BTA) were identified in CAS (above the redline in Figure 3), as reported in Table 2. The higher chromosomal peaks were identified on BTA5, BTA6, BTA19 and BTA28.

One thousand three hundred and fifty-five SNPs are involved in the definition of TOP_ROH and the summary indication of their annotated position on genome are shown in Figure S3. According to the Ensembl VEP tool, the SNPs are mapped in the intergenic (57.5%) and intragenic (42.5%) positions (Figure S3). If we consider the intragenic SNPs, the highest number of homozygote SNPs (≥25) was annotated within the *KHDRBS2* (*n* = 33), *CTNNA3* (*n* = 32), *ADGRL3* (*n* = 27), *CCSER1* (*n* = 26) and *CACNA2D1* (*n* = 25) genes. In addition, three homozygote SNPs were annotated in missense positions of the *PCDHA13* (Hapmap42803-BTA-110000-BTA7), *KRBA2* (BovineHD1900008383-BTA19) and *DNA2* (BTB-00981633-BTA28) genes.

In VBP, the genome regions that were the richest in TOP_ROH were identified on 13 autosomes, mainly on BTA6 and BTA23 (Table 3 and above the redline in Figure 3). These 17 TOP_ROH were defined by 1402 SNPs, of which 66% were in the intergenic and 33% in the intragenic positions (Figure S3). The highest number of homozygotes and intragenic SNPs (>25) were annotated within *KHDRBS2* (*n* = 33) and *CTNNA3* (*n* = 32), as for CAS. Missense SNP positions were annotated also in the VBP breed: *MAP3K19* (BovineHD0200018075–BTA2), *CYP4F2* (BovineHD0700002200–BTA7), *KRBA2* (BovineHD1900008383–BTA19) and *DNA2* (BTB-00981633-BTA28).

The proper TOP_ROH of VRP were 11, identified on 8 chromosomes (Table 4 and above the redline in Figure 3). SNPs delineating the TOP_ROH were 1304, annotated both in the intergenic (69.3%) and intragenic (30.8%) positions (Figure S3). Among the latter, the three missense SNP positions were in the *MAP3K19* (BovineHD0200018075–BTA2), *CFAP221* (ARS-BFGL-NGS-16745–BTA2) and *GALNT6* (ARS-BFGL-NGS-110943–BTA5) genes. The highest number of homozygous SNPs were harbored within the *THSD7B* (*n* = 48) and the *KHDRBS2* (*n* = 33) genes.

Within the CAS, VBP and VRP’s TOP_ROH, a total of 312, 212 and 162 genes were annotated, respectively. Among them, 6 genes (*C5H12orf50, C5H12orf29, CEP290, TMTC3, KITLG* and *KHDRBS2*) lied within the three regions in common among the three breeds (CAS–VBP–VRP). In addition, other 12 TOP_ROH were shared by two breeds: *n* = 5, CAS–VBP; *n* = 4, VBP–VRP; and *n* = 3, CAS–VRP.

**Figure 4:**
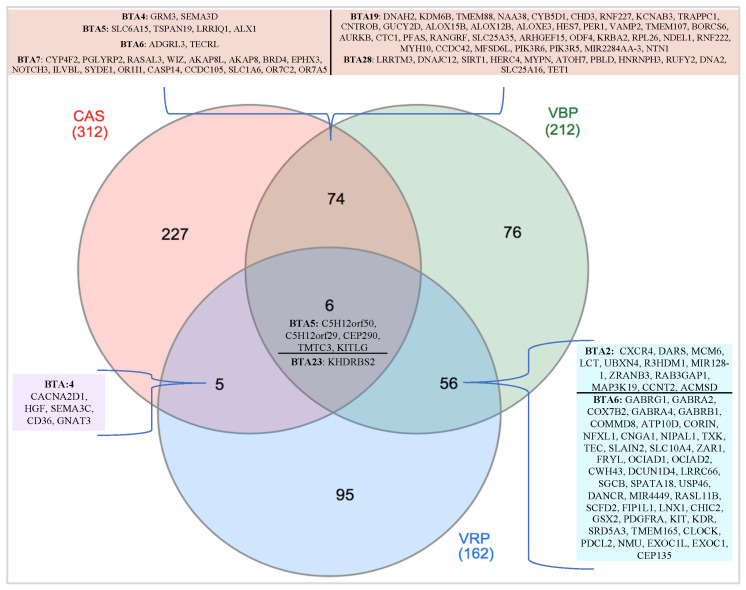
Venn diagram of the genes annotated in the TOP_ROH of CAS, VBP and VRP.

Figure 4 represents the Venn diagram of the proprietary and shared genes annotated in the TOP_ROH; for the common regions, the lists of genes are also reported. For CAS and VBP, a larger proportion of the shared genes was identified (*n* = 80, including the genes in common with VRP) with respect to CAS_VRP and VBP_VRP, while the lowest one was between CAS and VRP (*n* = 11). Not all common TOP_ROH harbored genes. The annotation, performed with the DAVID database, is presented in Supplementary Table S2.

The Table 5 reports details of the QTL associated with bovine traits (within the TOP_ROH) according to the nomenclature available in the AnimalQTLdb database [23].

### 3.4. Inbreeding Coefficients

**Figure 5:**
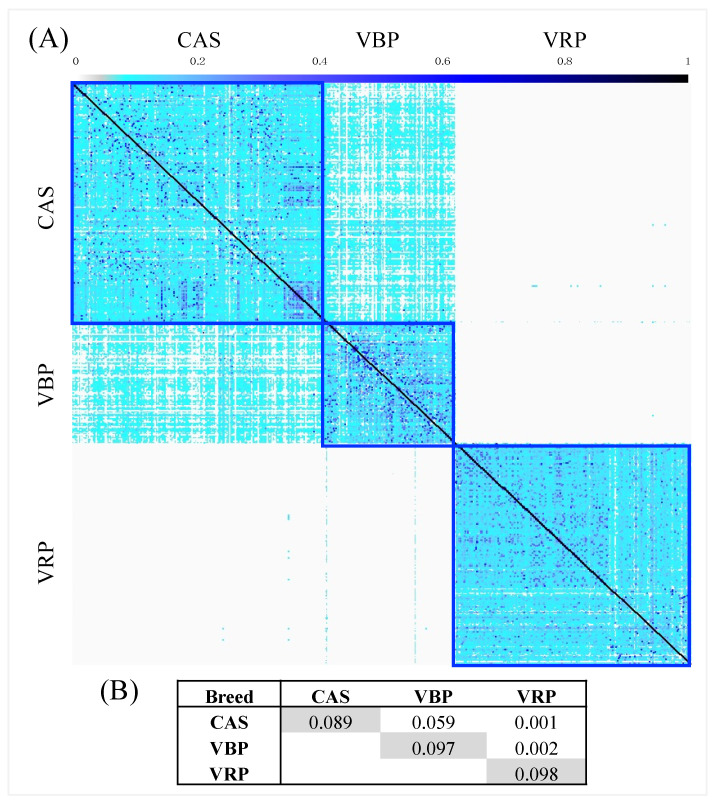
(**A**) Probability of alleles identical by descent (IBD) overview diagram calculated for all individuals. Blue squares represent the probability of IBD within each of the three populations; (**B**) average genomic relationships within (in diagonal—grey) and between populations (out of diagonal).

No sample duplication (pairwise IBD > 0.95) was identified for each breed. However, some samples belonging to CAS, VBP and VRP, with a genetic similarity greater than 50%, were found, suggesting a first-degree relationship between pairs of individuals (Figure 5A). The average genomic relationship within breed was similar in all populations, with a slightly higher value in VRP (Figure 5B). The average genomic relationship among breeds was higher between CAS and VBP. The VRP samples had a very low relationship with CAS and VBP.

The inbreeding coefficient, estimated using the proportion of homozygous SNPs distributed overall the autosomes SNP markers (*F*), were slightly negative (close to 0) in each population (min, max and mean values in Table 6). The average observed homozygote genotypes (67% in each breed), the expected homozygote ones and *F* are similar in CAS and VBP, and higher in VRP.

Individual F_ROH_ values varied from 0.002 (CAS) to 0.224 (VRP), with the highest average value for F_ROH_ (0.067) in the VRP (Table 6).

**Figure 6:**
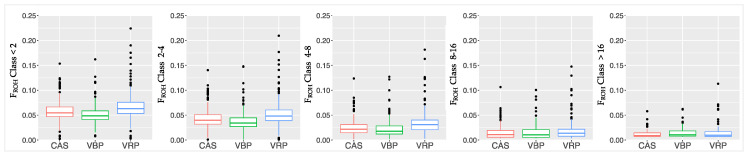
Boxplots built for each breed using the F_ROH_ values calculated within the five classes of ROH length (Mb).

As shown in Figure 6, the F_ROH_ values, calculated within each class of ROH length, differ among them: VRP samples showed clearly higher average F_ROH_ values in the three shortest classes of ROH length. F_ROH_ values reflect the ROH distribution and its average length across the classes. According to the same principle, differences in F_ROH_ were also found along all chromosomes (Figure S4).

**Figure 7:**
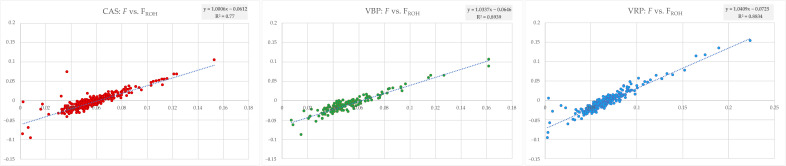
Regression and coefficient of determination (R^2^) calculated between *F* (y) and F_ROH_ (x) for each breed.

Among *F* and F_ROH_ exist a moderate-to-high correlation (CAS: R^2^ = 0.77; VBP: R^2^ = 0.89; VRP: R^2^ = 0.88), as shown in Figure 7.

## 4. Discussion

The three Aosta breeds have been sharing the same environment and farming practices for centuries. Nowadays, after the structuring of the breeding activities in herd books, even if they are part of the same herd book association, they are managed as three different populations with different selection indexes. A common denominator is that their milk is used for the Fontina cheese production, a DPO product that, in its manufacturing specifications, includes the rule that only the milk from Aosta cattle breeds can be used.

In term of selection, CAS and VBP are sharing a similar selection goal accounting for milk, meat and fighting ability. Differently, the dual-purpose selection of VRP is more oriented toward milk production [3]. The differentiation in selection goal occurred for decades and this may reflect the findings in term of genomic regions under selection that may be identified as the ROH.

Even if the three breeds share the same environment and farming structure, they appear to be genetically different. The genetic relationship between CAS and VBP, previously reported using microsatellite markers [25], was confirmed here with the use of SNP markers, as per the F_ST_ (both at population and at marker levels), PCA and ADMIXTURE results.

The F_ST_ statistic at the population level showed a value of genetic differentiation among the three breeds, which was around 0.05 when the comparison (both) involved VRP, but a lower one (F_ST_ = 0.019) between CAS and VBP. At the single-marker level, no differentiated SNPs (F_ST_ > 0.5) were identified in the CAS_VBP breed comparison, but several ones were found for the comparison of VRP with the other two breeds. These different values of F_ST_ can be affected by the origin of the three Aosta breeds: as known by historical evidences, the origin of VRP is independent from VBP and CAS. In fact, historical findings indicate that VRP was introduced in the area by the Burgundians in the 5th century AC.

It is interesting to mention that these genomic regions are harboring genes involved in the mammalian circadian rhythms regulation (*CLOCK*) [26], feed efficiency and growth traits (*CORIN*) [27], marbling (*LNX1*) [28] and immune response to mammary gland inflammation (*CBFA2T3*) [29]. It is worth noting that the VRP_CAS comparison identified the *KIT* and *PDGFRA* genes involved in melanogenesis and in coat color (spotting) (*KIT*) [30], as well as in intramuscular adipocyte development and marbling fat deposition (*PDGFRA*) [31], respectively. The coat color and shape (uniform and chestnut in CAS and red and white in VRP) is among the distinctive criteria of the breeds, whereas the differences in fat deposition can be ascribed to the peculiar constitution of the CAS breed. The vitality and attitude for dominance of this breed are a part indeed of a pretty “masculine” phenotype of CAS cows, showing curly hair and a large neck with developed anterior muscle masses, like the neighboring Hérens breed [32,33].

The genetic distinctness of the three populations is supported also by the PCA, showing clearly that the three breeds cluster separately: VBP and CAS are both placed on the same spatial position according to PC_1 (Figure 1A), explaining 41% of the total variance, and both separated from VRP. PC_2 is on the other hand separating the two breeds in two differentiated groups. The ADMIXTURE results also clearly support the evidence that the three breeds have distinct genetic origins.

The ROH analysis is particularly interesting in these three populations as it allows to disclose recent inbreeding caused by the managing of the populations, for example, by performing artificial insemination and with structured breeding plans, even if the selection scheme was carefully evaluated and planned to minimize the increasing of inbreeding [4].

The recent inbreeding’s loop produces small numbers of long ROH, influencing the sum of ROH much more than the total number of ROH itself. In the VRP, the regions linked to recent inbreeding are more frequent than the ones found in the other two breeds. In VRP, in fact, an ROH > 16 Mb has been found in 34% of samples (of which 28 animals had an ROH longer than 30 Mb), while in CAS and VBP, it was found in 26% and 30% of animals, respectively. Among the latter, 15 CAS and 12 VBP individuals had an ROH longer than 30 Mb.

Although a similar proportion of homozygous SNPs was identified among the three breeds (around 67%—Table 6), a higher number of homozygous SNPs concentrated in the ROH was detected in VRP (10.12%—calculated as the number of SNPs defining ROH/observed homozygotes). In CAS and in VBP, the proportion of homozygous SNPs mapping within the ROH is lower: 8.8% and 8.04%, respectively.

In VRP, a higher number of ROH and a longer size with respect to CAS and VBP were identified, mainly as a consequence of no introduction of animals from other regions. This is not the case for CAS, who has been recently recognized as genetically similar to the Hérens breed. The possibility to enroll progeny of the two breeds in the studbook of any of them was recently approved by the two breeder’s associations. This recent advance in reciprocal recognition occurred after some generation of known exchange of reproducers. In fact, already in 1929 [34], the closeness of CAS with Hérens was highlighted and the author already raised the hypothesis that the two breeds could be recognized genetically as one population. A previous study on microsatellites, carried out on the most important cattle breeds of the Alpine arc [25], also support the strong relationship between these two breeds. Before the approval of using Hérens bulls for breeding, crossbred mating was, however, already occurring between the CAS and VBP breeds, contributing to maintain the average level of inbreeding lower than in VRP (both *F* and F_ROH_).

The ROH are not randomly distributed across the genome and there are regions with a high prevalence of ROH. In the CAS breed, on the BTA5 located around 79–80 Mb, the highest number of samples (*n* = 94—31%) that shared the same TOP_ROH was identified. This TOP_ROH, defined by 18 homozygous SNPs (of which 5 are intronic), lies within the *TECRL* gene encoding for an enzyme involved in chemical reactions and pathways involving lipids, also reported to possibly play a role in puberty and female fertility in cattle [35]. In CAS, within the TOP_ROH on BTA4 11, the genes belong to the Homeobox family genes, of which *HOXA13, HOXA11, HOXA10, HOXA9, HOXA7, HOXA5, HOXA3* and *HOXA4* are involved in the reproductive tract and in development and fertility in males and females [36,37,38]. It interesting to note that a negative genetic relationships occurs between fighting ability and fertility, as observed in CAS [33], and a reduction in fertility has been found in Hérens cows, also empirically, for fighting ability across time [39]. The breed has no problems with fertility, like all Aosta breeds, well known for their hardiness, but a strong selection towards fighting ability; disregarding fitness characteristics could, in the long term, have a detrimental effect on fertility.

The second highest peak (TOP_ROH) found in CAS is located on chromosome 5 (max number of samples = 78, 25.4%), where the *KITLG* maps. This gene was included in regions under selection in cattle breeds [40,41] and is responsible for the coat color phenotype in different species [42,43]. In VBP, two higher peaks were identified on BTA6 and BTA23, where the maximum number of samples (*n* = 59, 38.5%) and (*n* = 45, 29.4%), respectively, shared intergenic and intragenic (*KHDRBS2* gene) homozygous regions. The *KHDRBS2* gene has been associated with fertility traits in goats [44] and in Brahman cows [45], and more recently with adaptability traits in Colombian cattle breeds [46]. This finding may relate to the VBP as well as other Aosta breeds, which are well adapted to harsh environmental conditions, such as the alpine pastures farming. Lastly, the regions shared by a higher number of VRP samples (more than half) are those found on chromosomes 2 (*n* = 150, 53.6%), 5 (*n* = 157, 56%) and 6 (*n* = 180, 64.3%). Except for the most representative regions on BTA2, for which an SNP maps in the intronic position of the *DARS1* gene, the other ones include SNPs lying closely to the *KITLG* (BTA5) and *KIT* (BTA6) genes, respectively.

We would like to underline the impact of selection on highly homozygous regions: several interesting genes in TOP_ROH include a large number of SNPs annotated within the gene itself. Although this information may be affected by the density of SNPs on the chip and by the length of the genes, it would be worth mentioning them. The *THSD7B* gene of the VRP’s TOP_ROH has 48 intronic SNPs annotated (Table S3), with an average distance among them of 17.7 kb, which is consistent with the average marker distance for this SNP chip spacing of approximately 19 kb (https://genomics.neogen.com/en/ggp-hd150k-dairy). For this gene, classified as an integral component of membranes (GO:0016021), no association study, at the best of our knowledge, is available. Another example is represented by the *KHDRBS2* gene on BTA23 (described above in the text), in which 33 homozygous intronic SNPs are annotated for all the three Aosta breeds at an average distance of 20.5 kb. Lastly, *CTNNA3* (*n* = 32 intronic SNPs spacing 20 kb), both in CAS and VBP, and *ADGRL3* (*n* = 27 intronic SNPs spacing 20.2 kb), *CCSER1* (*n* = 26 intronic SNPs spacing 17.9 kb) and *CACNA2D1* (*n* = 25 intronic SNPs plus a synonymous variant spacing 18.8 kb) in CAS are genes mostly annotated with homozygous SNPs. These genes were previously shown to be under positive selection and associated with marbling score in Korean cattle (*CTNNA3*) [47], with protein yield and percentage (*ADGRL3*) [48], with aggressiveness during gestation (*CCSER1*) [49] and with carcass and meat quality traits in the cattle (*CACNA2D*1) [50]. This is in line with the selection performed in the three Aosta breeds.

In VRP and VBP, a homozygous cluster on BTA6, involving GABA-A receptor subunit genes (*GABRA2, GABRA4, GABRB1* and *GABRG1*), was identified. These genes being the major inhibitory neurotransmitters in the mammalian brain, mediating anxiolytic activity and playing a key role in emotional and behavioral control in humans [51,52]. Even if speculative, we may comment that the fact that this region does not appear to be in autozygosity in CAS may lead to variability in expression regarding the behavior of cows and for the specific selection operated by the farmers on this breed for the “*Battailes des Reines*”. We may assume that, during the non-ferocious match, the reaction to the visual and physical view of the opponent may be mediated by these groups of genes. The behaviors exhibited during the matches is the same that cows show in pastures when unfamiliar individuals from different herds meet at the beginning of the summer season.

## 5. Conclusions

This study, performed on the three Aosta breeds (Aosta Red Pied, Aosta Black Pied and Aosta Chestnut) on a large number of individuals and on a medium density SNP chip (150K), is indicating that farmers in their mating decisions still prioritized adaptation to the environment and thus the farming system envisaging summer pasture, a practice that have characterized these breeds for centuries. All three breeds were shown to have a large number of regions in autozygosity, harboring genes that appear to be linked to efficiency and functional traits, i.e., characteristics for adapting to the environment. As such, with respect to other populations, e.g., Holstein, where breeding plans have strongly acted on genomic regions linked to milk production, the genomic status of these populations shows that, for decades, breeders pursued among the selection objectives the adaptation to the environment, making these breeds resilient and efficient producers in a changing environment. The low extent of recent autozygosity found in this study is a tangible measure of the success of the reproductive scheme to implement selection in the Aosta breeds, as pursued in the last 30 years, aimed also at the maintenance of the genetic variability in these three breeds.

## Figures and Tables

**Table 1:** Runs of Homozygosity (ROH) descriptive statistics; length expressed in bp.

Breed	Tot. ROH	Min (n)	Max (n)	Mean (n)	Min Length	Max Length	Mean Length
**CAS**	14,921	4	86	50	1,000,152	39,512,804	2,872,455
**VBP**	6875	10	78	45	1,002,352	55,392,599	2,931,795
**VRP**	14,604	4	90	53	1,000,089	71,168,012	3,149,324

**Table 2:** CAS TOP_ROH and annotated genes.

Chr	Start	End	Length	Genes
9801	94,343,951	94,422,954	79,003	SPATA16
4	33,711,848	35,755,375	2,043,527	GRM3, SEMA3D
4	37,970,282	40,696,202	2,725,920	CACNA2D1, HGF, SEMA3C, CD36, GNAT3
4	45,618,675	47,116,336	1,497,661	LHFPL3, KMT2E, SRPK2, PUS7, RINT1, EFCAB10, ATXN7L1, CDHR3, MIR2284B
4	67,374,218	68,976,979	1,602,761	CREB5, JAZF1, TAX1BP1, HIBADH, EVX1, HOXA13, HOXA11, HOXA10, MIR196B, HOXA9, HOXA7, HOXA6, HOXA5, HOXA3, HOXA4, HOXA2, HOXA1
5	13,970,721	15,271,475	1,300,754	SLC6A15, TSPAN19, LRRIQ1, ALX1
5	17,066,241	19,211,885	5,241,164	C5H12orf50, C5H12orf29, CEP290, TMTC3, KITLG,
5	64,842,324	66,224,046	1,381,722	ANO4, MIR2434, SLC5A8, UTP20, ARL1, SPIC, MYBPC1, CHPT1, SYCP3, GNPTAB, DRAM1, WASHC3, NUP37, PARPBP, PMCH, IGF1
6	34,018,647	35,004,065	985,418	MMRN1, SNCA
6	76,546,983	81,022,352	4,475,369	ADGRL3, TECRL
6	90,365,492	91,722,935	1,357,443	THAP6, DAPH, CDKL2, G3BP2, USO1, PPEF2, NAAA, SDAD1, CXCL9, ART3, CXCL10, CXCL11, NUP54, SCARB2, FAM47E, STBD1, CCDC158, SHROOM3
7	76,951,29	9,853,485	2,158,356	CYP4F2, PGLYRP2, RASAL3, WIZ, AKAP8L, AKAP8, BRD4, EPHX3, NOTCH3, ILVBL, SYDE1, OR1I1, CASP14, CCDC105, SLC1A6, OR7C2, OR7A5, OR7A17
7	49,571,781	52,828,781	3,257,000	NME5, BRD8, KIF20A, CDC23, GFRA3, CDC25C, SLBP2, FAM53C, MIR2459, KDM3B, REEP2, EGR1, ETF1, HSPA9, CTNNA1, LRRTM2, SIL1, SNHG4, MATR3, PAIP2, SLC23A1, MZB1, PROB1, SPATA24, DNAJC18, ECSCR, SMIM33, TMEM173, UBE2D2, CXXC5, PSD2, NRG2, PURA, IGIP, CYSTM1, PFDN1, HBEGF, SLC4A9, NKHD1, EIF4EBP3, SRA1, APBB3, SLC35A4, CD14, TMCO6, NDUFA2, IK, WDR55, DND1, HARS, HARS2, ZMAT2, PCDHA13, PCDHA3, PCDHB1, PCDHB8, PCDHB14, PCDHB11, SLC25A2, TAF7, PCDHGA2, PCDHGB4, PCDHGA8, PCDHGC3, DIAPH1, HDAC3, RELL2, FCHSD1, ARAP3
14	52,337,429	52,854,862	517,433	-
19	26,858,526	28,789,212	1,930,686	ASGR2, ASGR1, DLG4, ACADVL, MIR324, DVL2, PHF23, GABARAP, CTDNEP1, ELP5, CLDN7, SLC2A4, YBX2, EIF5A, GPS2, NEURL4, ACAP1, KCTD11, TMEM95, TNK1, PLSCR3, TMEM256, NLGN2, SPEM1, SPEM2, TMEM102, FGF11, CHRNB1, ZBTB4, SLC35G6, POLR2A, TNFSF12, TNFSF13, SENP3, EIF4A1, CD68, MPDU1, SOX15, FXR2, SAT2, SHBG, ATP1B2, TP53, WRAP53, EFNB3, DNAH2, KDM6B, TMEM88, NAA38, CYB5D1, CHD3, RNF227, KCNAB3, TRAPPC1, CNTROB, GUCY2D, ALOX15B, ALOX12B, ALOXE3, HES7, PER1, VAMP2, TMEM107, BORCS6, AURKB, CTC1, PFAS, RANGRF, SLC25A35, ARHGEF15, ODF4, KRBA2, RPL26, NDEL1, RNF222, MYH10, CCDC42, MFSD6L, PIK3R6, PIK3R5, MIR2284AA-3, NTN1, STX8
19	33,207,693	34,981,210	1,773,517	TRPV2, UBB, CENPV, PIGL, NCOR1, TTC19, ZSWIM7, ADORA2B, SPECC1, AKAP10, ULK2, ALDH3A1, SLC47A2, ALDH3A2, SLC47A1, RNF112, MFAP4, MAPK7, B9D1, EPN2, GRAP, SLC5A10, FAM83G, PRPSAP2, SHMT1, SMCR8, TOP3A, MIEF2, FLII, LLGL1, ALKBH5, MYO15A, DRG2, GID4, ATPAF2, DRC3, TOM1L2, SREBF1, MIR33B, RAI1, PEMT, RASD1, MED9, NT5M, COPS3, FLCN, PLD6, MPRIP
23	26,021	1,616,849	1,590,828	KHDRBS2
28	23,475,117	25,022,068	1,546,951	LRRTM3, DNAJC12, SIRT1, HERC4, MYPN, ATOH7, PBLD, HNRNPH3, RUFY2, DNA2, SLC25A16, TET1

**Table 3:** VBP TOP_ROH and annotated genes.

Chr	Start	End	Length	Genes
1	31,843,014	32,866,434	1,023,420	CADM2
1	63,602,126	66,515,809	2,913,683	IGSF11, C1H3orf30, UPK1B, B4GALT4, ARHGAP31, TMEM39A, POGLUT1, TIMMDC1, CD80, ADPRH, PLA1A, POPDC2, COX17, MAATS1, NR1I2, GSK3B, MIR6529B, MIR6529A, GPR156, LRRC58, FSTL1, NDUFB4, HGD, RABL3, GTF2E1, STXBP5L, POLQ, ARGFX, FBXO40, HCLS1, GOLGB1, IQCB1, EAF2, SLC15A2, ILDR1
2	60,571,566	64,263,556	3,691,990	CXCR4, DARS, MCM6, LCT, UBXN4, R3HDM1, MIR128-1, ZRANB3, RAB3GAP1, MAP3K19, CCNT2, ACMSD, TMEM163, MGAT5
3	96,013,420	96,080,539	67,119	ELAVL4
4	33,711,848	36,320,534	2,608,686	GRM3, SEMA3D, SEMA3A
5	13,838,215	15,271,475	1,433,260	SLC6A15, TSPAN19, LRRIQ1, ALX1
5	17,157,155	18,735,088	1,577,933	C5H12orf50, C5H12orf29, CEP290, TMTC3, KITLG,
6	64,151,594	71,501,595	7,350,001	GABRG1, GABRA2, COX7B2, GABRA4, GABRB1, COMMD8, ATP10D, CORIN, NFXL1, CNGA1, NIPAL1, TXK, TEC, SLAIN2, SLC10A4, ZAR1, FRYL, OCIAD1, OCIAD2, CWH43, DCUN1D4, LRRC66, SGCB, SPATA18, USP46, DANCR, MIR4449, RASL11B, SCFD2, FIP1L1, LNX1, CHIC2, GSX2, PDGFRA, KIT, KDR, SRD5A3, TMEM165, CLOCK, PDCL2, NMU, EXOC1L, EXOC1, CEP135
6	75,035,526	80,484,712	5,449,186	ADGRL3, TECRL
7	7,513,855	8,905,045	1,391,190	CYP4F2, PGLYRP2, RASAL3, WIZ, AKAP8L, AKAP8, BRD4, EPHX3, NOTCH3, ILVBL, SYDE1, OR1I1, CASP14, CCDC105, SLC1A6, OR7C2, OR7A5
8	3,8571,728	39,680,225	1,108,497	IL33, RANBP6, KIAA2026, MLANA, ERMP1, RIC1, PDCD1LG2, CD274, PLGRKT, INSL6, JAK2, RCL1
8	86,700,245	86,837,166	136,921	-
13	53,727,128	54,052,028	324,900	MYT1, NPBWR2, OPRL1, LKAAEAR1, RGS19, TCEA2, SOX18, C13H20orf204, PRPF6, SAMD10, ZNF512B, UCKL1, MIR1388, DNAJC5, TPD52L2, ABHD16B, ZBTB46
19	27,455,006	28,468,488	1,013,482	DNAH2, KDM6B, TMEM88, NAA38, CYB5D1, CHD3, RNF227, KCNAB3, TRAPPC1, CNTROB, GUCY2D, ALOX15B, ALOX12B, ALOXE3, HES7, PER1, VAMP2, TMEM107, BORCS6, AURKB, CTC1, PFAS, RANGRF, SLC25A35, ARHGEF15, ODF4, KRBA2, RPL26, NDEL1, RNF222, MYH10, CCDC42, MFSD6L, PIK3R6, PIK3R5, MIR2284AA-3, NTN1
20	36,530,066	37,722,404	1,192,338	GDNF, WDR70, NUP155, MIR2360, CPLANE1, NIPBL, SLC1A3
23	26,021	1,674,058	1,648,037	KHDRBS2
28	23,475,117	24,940,953	1,465,836	LRRTM3, DNAJC12, SIRT1, HERC4, MYPN, ATOH7, PBLD, HNRNPH3, RUFY2, DNA2, SLC25A16, TET1

**Table 4:** VRP TOP_ROH and annotated genes.

Chr	Start	End	Length	Genes
2	58,568,475	62,445,218	3,876,743	SPOPL, HNMT, THSD7B, CXCR4, DARS, MCM6, LCT, UBXN4, R3HDM1, MIR128-1, ZRANB3, RAB3GAP1, MAP3K19, CCNT2, ACMSD
2	70,949,947	72,719,297	1,769,350	STEAP3, C2H2orf76, DBI, TMEM37, SCTR, CFAP221, TMEM177, PTPN4, EPB41L5, TMEM185B, RALB, INHBB, GLI2
4	38,505,994	40,696,202	2,190,208	CACNA2D1, HGF, SEMA3C, CD36, GNAT3
5	15,165,042	19,853,714	4,688,672	RASSF9, NTS, MGAT4C, C5H12orf50, C5H12orf29, CEP290, TMTC3, KITLG, DUSP6, POC1B, GALNT4, ATP2B1
5	27,596,480	29,299,759	1,703,279	KRT81, KRT7, C5H12orf80, KRT80, ATG101, NR4A1, GRASP, ACVR1B, ACVRL1, ANKRD33, FIGNL2, SCN8A, SLC4A8, GALNT6, CELA1, BIN2, SMAGP, DAZAP2, POU6F1, TFCP2, CSRNP2, LETMD1, SLC11A2, HIGD1C, METTL7A, TMPRSS12, ATF1, DIP2B
6	60,191,863	71,910,070	11,718,207	BEND4, SHISA3, ATP8A1, GRXCR1, KCTD8, YIPF7, GUF1, GNPDA2, GABRG1, GABRA2, COX7B2, GABRA4, GABRB1, COMMD8, ATP10D, CORIN, NFXL1, CNGA1, NIPAL1, TXK, TEC, SLAIN2, SLC10A4, ZAR1, FRYL, OCIAD1, OCIAD2, CWH43, DCUN1D4, LRRC66, SGCB, SPATA18, USP46, DANCR, MIR4449, RASL11B, SCFD2, FIP1L1, LNX1, CHIC2, GSX2, PDGFRA, KIT, KDR, SRD5A3, TMEM165, CLOCK, PDCL2, NMU, EXOC1L, EXOC1, CEP135, KIAA1211, AASDH, PPAT, PAICS, SRP72, ARL9, THEGL
6	75,082,684	76,679,327	1,596,643	-
8	84,984,551	88,740,000	3,755,449	PHF2, BARX1, PTPDC1, LOC112447831, MIRLET7A-1, MIRLET7F-1, MIRLET7D, ZNF169, SPTLC1, ROR2, NFIL3, AUH, SYK, DIRAS2, GADD45G, SEMA4D
11	59,219,240	60,949,119	1,729,879	C11H2orf74, AHSA2, USP34, XPO1, FAM161A, CCT4, COMMD1, B3GNT2
15	15,878,790	16,444,199	565,409	PIWIL4, FUT4, C15H11orf97, CWF19L2, GUCY1A2
23	26,021	1,674,058	1,648,037	KHDRBS2

**Table 5:** Details of the QTL associated with bovine traits.

QTL General Classification	QTL Specific Classification	Count of QTL		
		CAS	VBP	VRP
Exterior Traits	Behavioral	0	3	2
	Coat texture	0	7	0
	Conformation	16	25	19
	Limb traits	11	24	18
	Pigmentation	16	55	43
	Udder traits	20	38	33
Health Traits	Disease	10	13	15
	General health parameters	1	0	0
	Mastitis	12	15	11
	Parasite/pest resistance	1	1	0
Meat and Carcass Traits	Anatomy	13	20	18
	Chemistry	3	8	5
	Fatness	2	6	3
	Fatty acid content	0	2	1
	Sensory characteristics	3	1	1
Milk Traits	Milk composition—fat	73	55	49
	Milk composition—other	3	10	22
	Milk composition—protein	1646	1916	1216
	Milk processing trait	54	35	11
	Milk yield	19	18	6
Production Traits	Feed intake	4	8	3
	Growth	73	92	146
	Life history traits	10	15	12
	Lifetime production	49	54	13
Reproduction Traits	Fertility	54	41	42
	General reproduction parameters	8	11	5
	Semen quality	1	2	1

**Table 6:** Descriptive statistics for *F* and F_ROH_.

Breed	*F*					F_ROH_		
	Obs Hom ^1^	Exp Hom ^2^	Min	Max	Mean	Min	Max	Mean
CAS	56,657.4	56,741.8	−0.096	0.104	−0.003	0.002	0.153	0.058
VBP	56,562.7	56,823.4	−0.089	0.105	−0.010	0.008	0.162	0.053
VRP	56,943.6	57,025.9	−0.096	0.153	−0.003	0.003	0.224	0.067

^1^ Observed homozygotes; ^2^ Expected homozygotes.

## Supplementary Materials

The following are available online at https://www.mdpi.com/2076-2615/10/12/2385/s1, Figure S1: Manhattan plot of marker-based F_ST_ values obtained in each breed comparison, Figure S2: Frequencies of ROH per chromosomes and chromosomes mean length (Mb) calculated for the three breeds, Figure S3: Annotation of SNPs defining the three breed TOP_ROH, Figure S4: F_ROH_ along all chromosomes of the three breeds, Table S1: F_ST_ by marker values for CAS vs. VRP (sheet1) and for VBP vs. VRP (sheet 2) comparisons, Table S2: Annotation of genes mapped in CAS TOP_ROH (sheet 1), in VBP TOP_ROH (sheet 2), and VRP TOP_ROH (sheet 3) according to DAVID online Database, Table S3: SNPs annotation in the TOP_ROH regions for the CAS, VBP and VRP breeds.
