# Supplementary figures and images for: The Genomic Variation in the Aosta Cattle Breeds Raised in an Extensive Alpine Farming System
Source: Animals (Basel). 2020 Dec 12;10(12):2385. doi: 10.3390/ani10122385 (PMC7764440; doi:10.3390/ani10122385)

# CAS

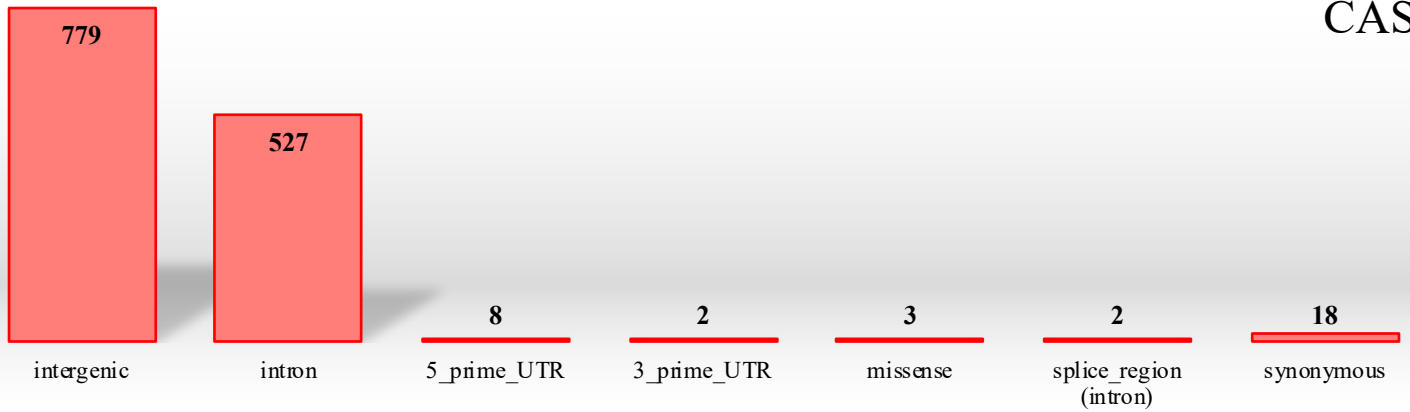

# VRP

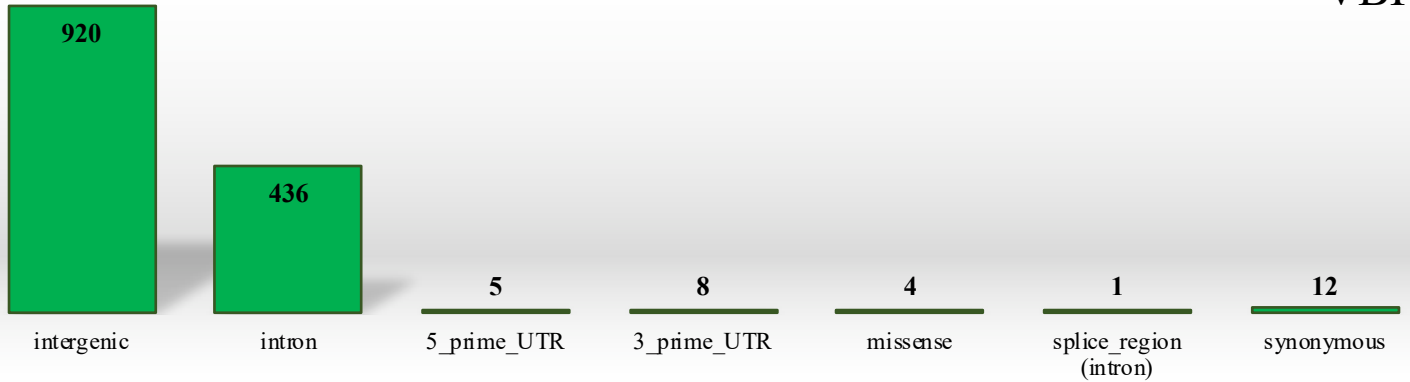

# VRP

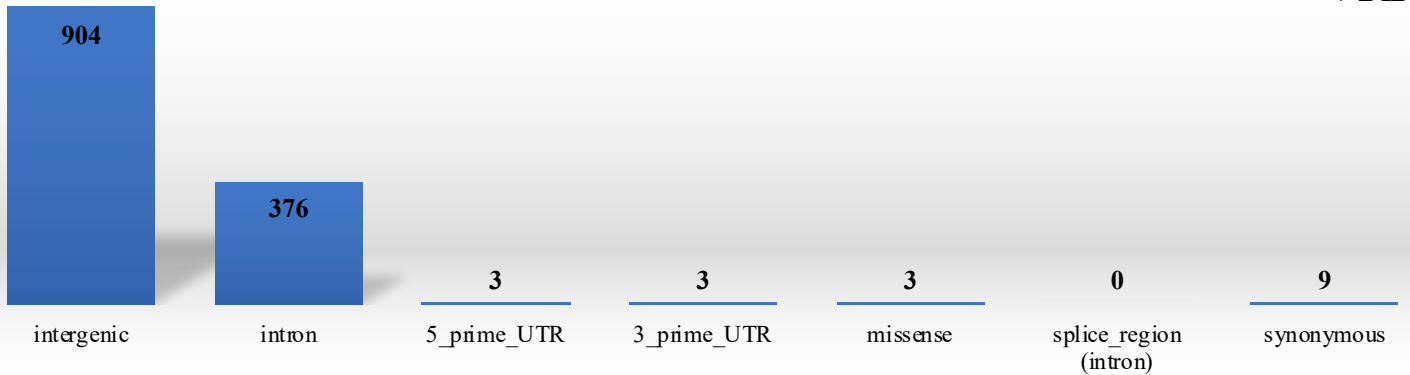

Supplement: Supplementary file 1 [file animals-10-02385-s001.zip › Supplementary/Figure_S3.pdf]

# CAS

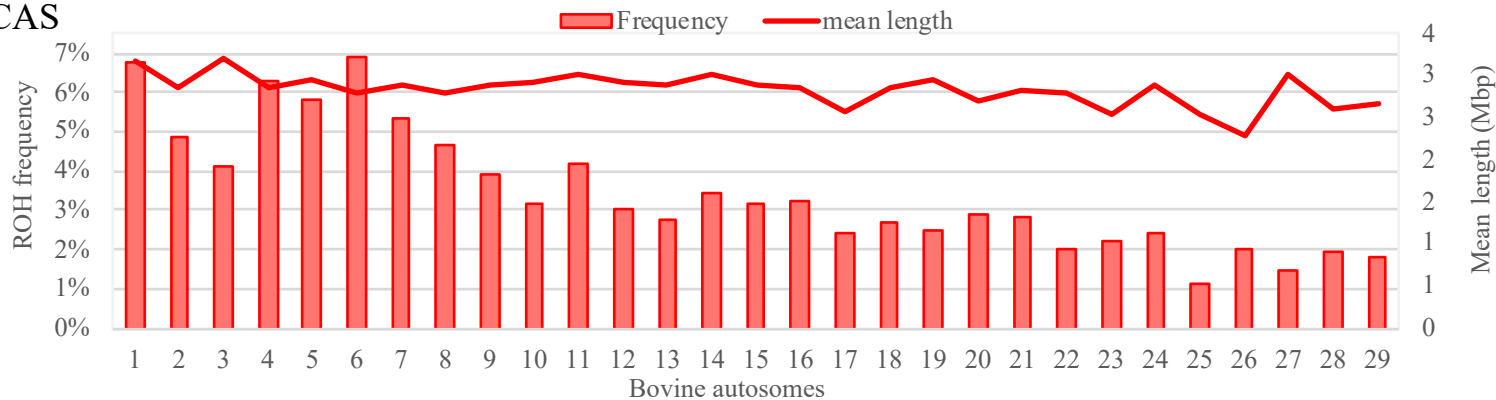

# VBP

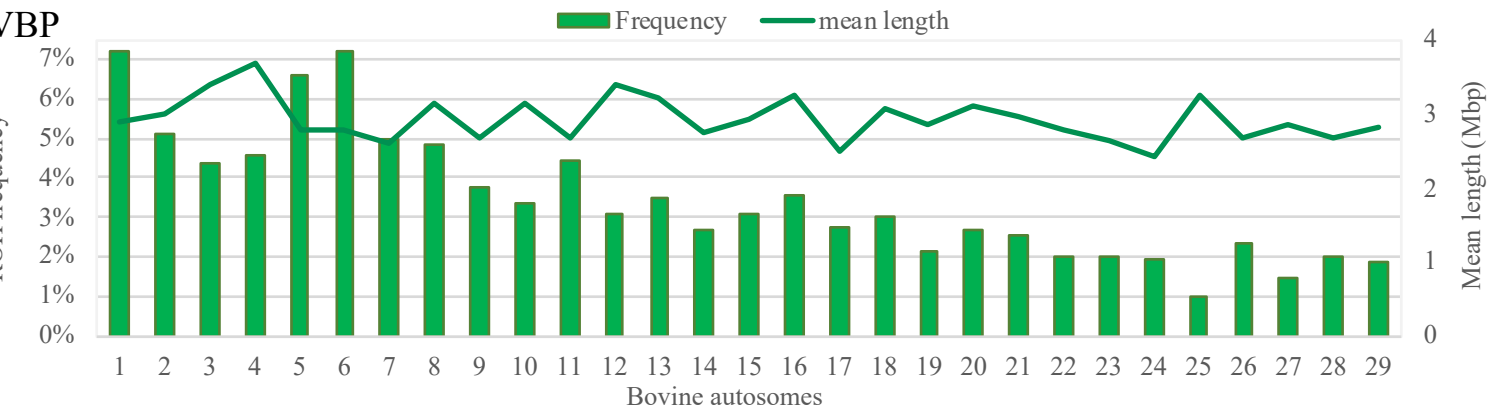

# VRP

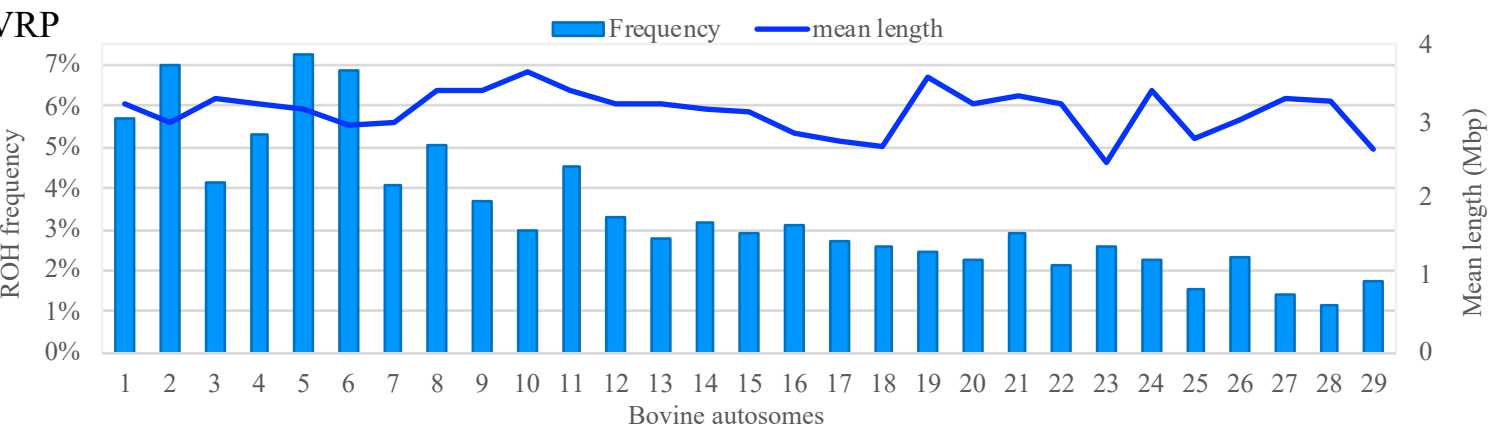

Supplement: Supplementary file 1 [file animals-10-02385-s001.zip › Supplementary/Figure_S2.pdf]

CAS-VBP

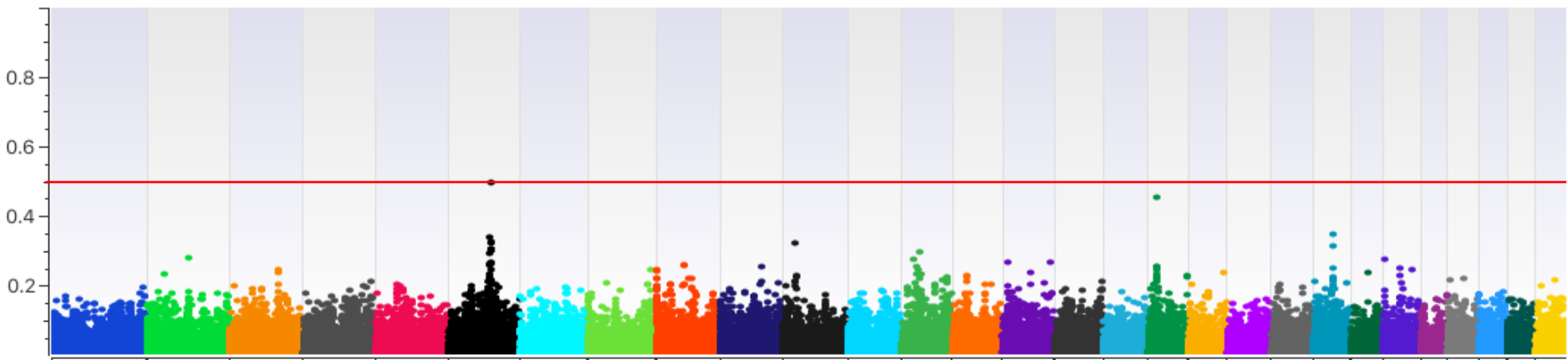

CAS-VRP

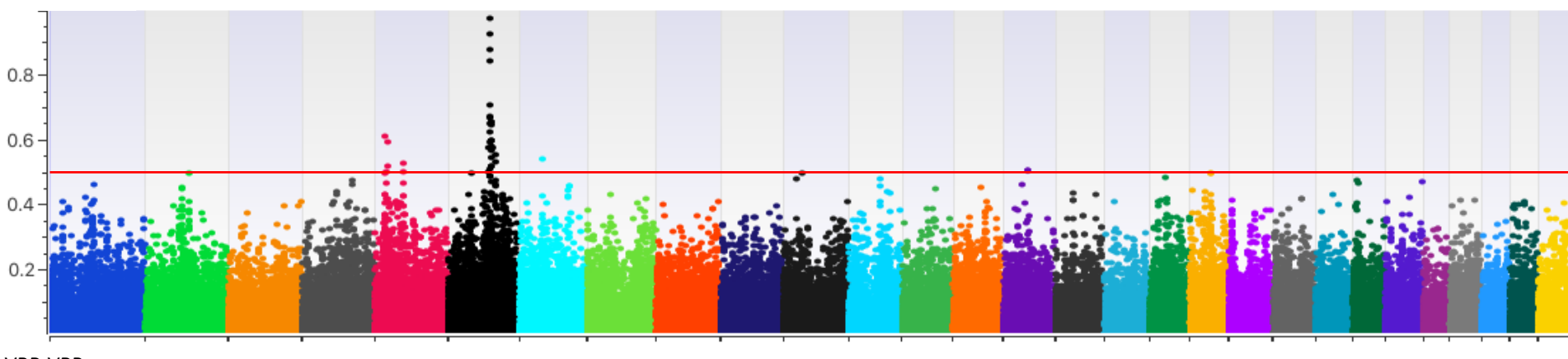

VBP-VRP

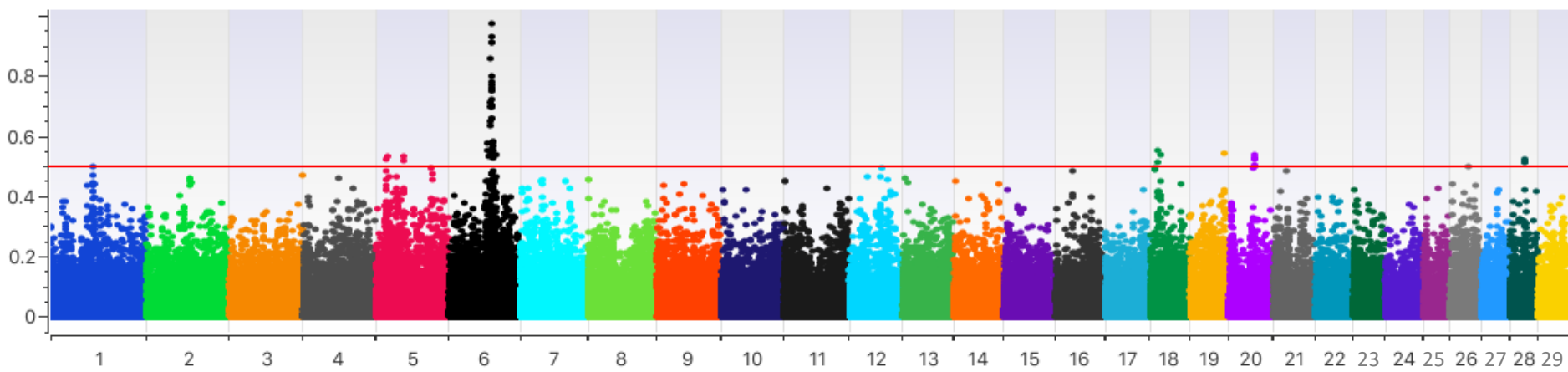

Supplement: Supplementary file 1 [file animals-10-02385-s001.zip › Supplementary/Figure_S1.pdf]

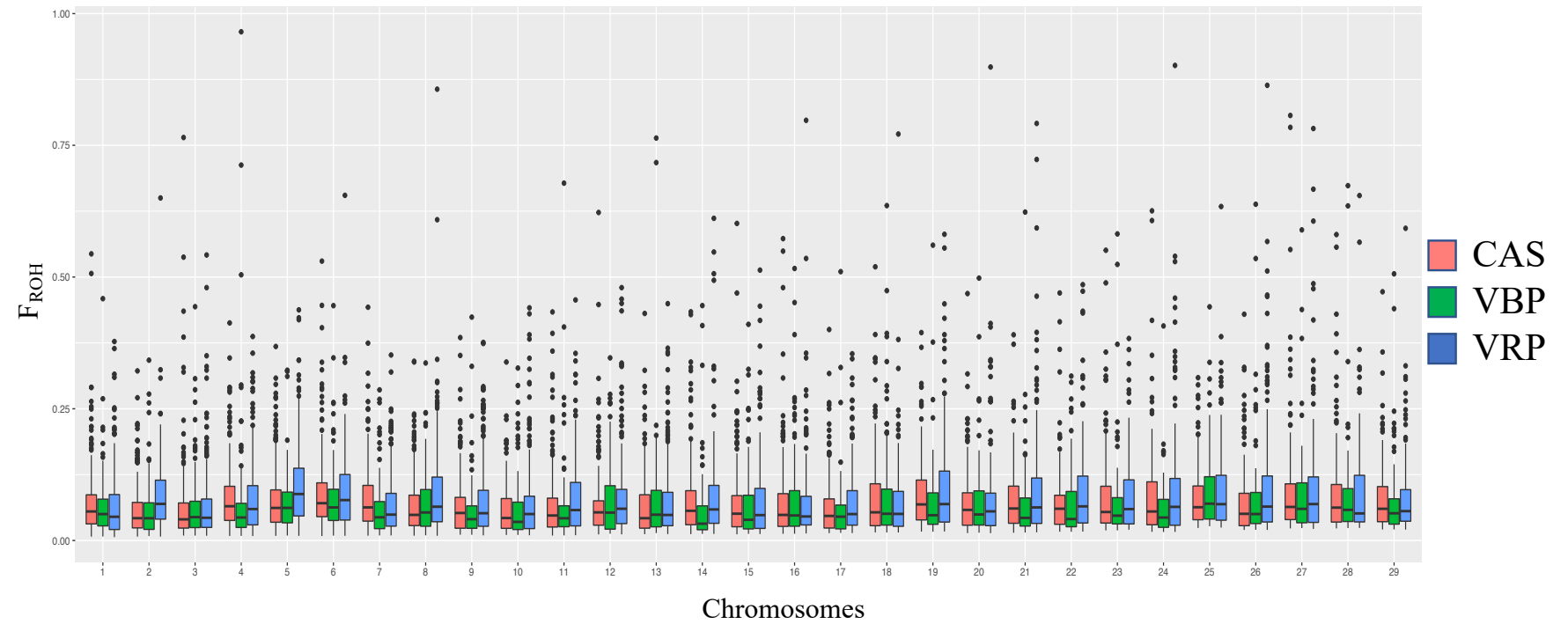

Supplement: Supplementary file 1 [file animals-10-02385-s001.zip › Supplementary/Figure_S4.pdf]
